# Presence of *Legionella* spp. in Hot Water Networks of Different Italian Residential Buildings: A Three-Year Survey

**DOI:** 10.3390/ijerph14111296

**Published:** 2017-10-26

**Authors:** Michele Totaro, Paola Valentini, Anna Laura Costa, Lorenzo Frendo, Alessia Cappello, Beatrice Casini, Mario Miccoli, Gaetano Privitera, Angelo Baggiani

**Affiliations:** 1 Department of Translational Research N.T.M.S., University of Pisa, 56126 Pisa, Italy; micheleto@hotmail.it (M.T.); paola.valentini@dps.unipi.it (P.V.); anna.costa@med.unipi.it (A.L.C.); lorenzo.frendo@hotmail.com (L.F.); ale.cappello294@libero.it (A.C.); beatrice.casini@med.unipi.it (B.C.); gaetano.privitera@med.unipi.it (G.P.); 2 Department of Clinical and Experimental Medicine, University of Pisa, 56126 Pisa, Italy; mario.miccoli@med.unipi.it

**Keywords:** *Legionella*, residential buildings, water risk, community-acquired Legionnaire’s disease cases

## Abstract

Although the European reports highlight an increase in community-acquired Legionnaires’ disease cases, the risk of *Legionella* spp. in private houses is underestimated. In Pisa (Italy) we performed a three-year survey on Legionella presence in 121 buildings with an independent hot water production (IB); 64 buildings with a central hot water production (CB); and 35 buildings with a solar thermal system for hot water production (TB). From all the 220 buildings *Legionella* spp. was researched in two hot water samples collected either at the recirculation point or on the first floor and on the last floor, while the potable water quality was analysed in three cold water samples collected at the inlet from the aqueduct network, at the exit from the autoclave, and at the most remote tap. *Legionella pneumophila* sg1, *Legionella pneumophila* sg2–16, and non-*pneumophila Legionella* species were detected in 26% of the hot water networks, mostly in CB and TB. In these buildings we detected correlations between the presence of *Legionella* and the total chlorine concentration decrease and/or the increase of the temperature. Cold water resulted free from microbiological hazards, with the exception of *Serratia liquefaciens* and *Enterobacter cloacae* isolated at the exit from two different autoclaves. We observed an increase in total microbial counts at 22 °C and 37 °C between the samples collected at the most remote taps compared to the ones collected at the inlet from the aqueduct. The study highlights a condition of potential risk for susceptible categories of population and supports the need for measures of risk assessment and control.

## 1. Introduction

*Legionella* spp. is a Gram-negative aerobic bacterium, which is widely present in soil and freshwater but that can also contaminate water systems; it replicates between 25 °C and 42 °C surviving at higher temperatures [1,2]. *Legionella* infection may cause distinct clinical diseases as Legionnaires’ Disease and Pontiac fever. Infection is acquired by inhalation, aspiration, or micro-aspiration of *Legionella*-carrying aerosols. Droplets carrying the pathogen can originate by water spraying or by gurgling air through contaminated water. Immunocompromisation, chronic diseases, and old age are predisposing factors for the development of the disease [3]. *Legionella pneumophila* serogroup 1 is the species most commonly associated with the disease. It is the cause of 95% of *Legionella* infections in Europe and 85% worldwide [4,5,6]. The surveillance of legionellosis is coordinated in Europe by the European Centre for Disease Prevention and Control (ECDC), and in Italy by the Italian National Institute of Health. The latest data, related to 2015, give 1553 cases of legionellosis in the Italian population, corresponding to a notification rate of 2.55 cases per million inhabitants. However, it must be noted that 2015 has seen an increase of 5.6% in the cases when compared with the previous year. Of the 1553 notified cases, 83 (5.3%) were hospital acquired, while 1221 (78.5%) were community-acquired infections, underscoring the relevance of *Legionella* contamination of community environment and the subsequent need for control [7,8]. Considering the high percentage of *Legionella* community-acquired infections, Italian and European literature data on *Legionella* contamination in air conditioning systems and hot water networks are available both for hospitals [9,10], and for small residential buildings [11].

There are several studies reporting *Legionella* presence in water not associated with a specific outbreak; they include studies from Italy [12], Germany [13], Turkey [14], Georgia [15], Denmark [16], Poland [17], etc.

In Italy, in 2015 a guideline for legionellosis control in all the settings has been updated by the Italian Ministry of Health [18]. This technical document gives indications for prevention and control of *Legionella* in hot water networks with instructions for Legionella risk assessment in different contexts. Moreover, the Council Directive 98/83/EC [19] identifies the importance and the responsibilities for water quality control in buildings. In a previous study, a survey on 81 Italian apartments buildings found the colonization of *Legionella* in 20% of hot water networks [20]. Following the previous published data, the aim of this study is the assessment of *Legionella* colonization in hot water networks in residential buildings in the Pisa district (Italy) in the period 2014–2017.

## 2. Materials and Methods

### 2.1. Setting and Inspections

Following the requests of some building administrators of controlling the drinking water quality, the survey was performed from April 2014 to April 2017 on 220 residential buildings located in Pisa (Italy). Buildings are situated in different areas (old town, new town, industrial area, countryside, sea district, etc.). We investigated buildings with old and with new water networks. The buildings were mainly of small sizes with a range between four and twenty flats. One-hundred twenty-one buildings had an independent hot water production (IB); 64 buildings had a central hot water production (CB); 35 buildings had a solar thermal system for hot water production (TB).

In each technical plant an inspection visit was performed with the aim of analysing the adequate functioning of the thermal and water power plants. Inspections were conducted following a checklist organized with some information such as name, address of building, type of hot water production system, presence of devices (recirculation, boilers, softeners, autoclaves, etc.), water disinfection system, periodicity and type of water system maintenance and cleaning, water supplying, and number of floors and apartments.

### 2.2. Sampling

From each water system three cold water samples were taken at the inlet from the aqueduct network into the building pipework (Point I); at the exit from pressure autoclave (point E); and at the most remote tap from the autoclave (point T). Therefore, 660 cold water samples were analysed for the determination of potability requirements as suggested by the Council Directive 98/83/EC [19].

At the same time two hot water samples were collected either at the recirculation point or on the first floor of the building (Point A) and on the last floor (Point B). Therefore, 440 hot water samples were collected for *Legionella* spp. detection as suggested by the Italian guidelines for legionellosis control.

Water temperature and total chlorine concentration, were determined in cold and hot water samples, while pH, and conductivity values were measured only in hot water samples.

### 2.3. Laboratory Tests

In accordance to the Council Directive 98/83/EC [19] total microbial count at 22 °C and at 37 °C, coliforms and enterococci counts were determined. Total microbial count was performed in Reasoner’s 2A agar (Oxoid Ltd., Basingstoke, Hampshire, UK) according to ISO6222:2001 [21]. Coliforms and enterococci bacteria were researched in 100 mL samples using Colilert 100 mL (Idexx, Westbrook, ME, USA) and Slanetz Bartley Agar (Biolife, Milan, Italy), respectively, according to ISO 9308-1:2012 [22] and ISO 7899-2:2003 [23]. Bacteria species confirmation of suspect colonies was obtained by Mini API galleries (bioMeriéux, Marcy l’Etoile, France).

The samples were tested for the presence of *Legionella* in accordance to the Italian guidelines and ISO 11731:1998 [24]. One litre of water was filtered through a 0.2 µm membrane (Millipore, Billerica, MA, USA), which was subsequently immersed in 10 mL of the same water and sonicated for 5 min, allowing the detachment of cells from the membrane and their suspension in water. Suspension was subjected to a thermal inactivation treatment at 50 °C for 30 min with the aim to select *Legionella* spp., inactivating all the microbial species not resistant to high temperature. Afterwards, 0.1 mL of the suspension was seeded in triplicate on Legionella selective medium (cefamandole, polymyxin B, anisomycin) (Oxoid Ltd., Basingstoke, Hampshire, UK) and the plates were incubated at 37 °C for 7–10 days within jars containing modified atmosphere (2.5% CO_2_). Suspected *Legionella* colonies grown on the medium were subjected to species and serogroup identification analysis using a multi-purpose latex agglutination test (Legionella Latex Test, Oxoid Ltd., Basingstoke, Hampshire, UK).

### 2.4. Statistical Analysis

The Shapiro–Wilk test was performed to verify normality of distributions. The Kruskall–Wallis test and the Dunn’s test were performed to compare the total microbial counts at 22 °C and at 37 °C detected in different sampling points. Power tests were used to estimate the sample sizes, the 1-β values of the significant variables were >0.8, assuring a low risk of type II error and an appropriate sample sizes. Correlation tests were performed, and Pearson’s coefficients were calculated with the aim to analyse the correlations between physical-chemical parameters of the samples (temperature, chlorine concentration, and conductivity) and the presence of *Legionella* spp. These tests were independently applied for IB, CB, and TB. Confidence levels of 95% were defined for the statistical tests. Therefore, we considered the following ranges of values: 0–0.3 (weak correlation); 0.3–0.7 (moderate correlation); 0.7–1 (strong correlation). All statistical analysis was carried out using the SPSS software package (version 17.0.1, IBM, Armonk, NY, USA).

## 3. Results

### 3.1. Inspections and Physical-Chemical Results

All 121 IB present an autoclave system which collects municipal water before being injected in water networks. Water is softened in 36 out of 121 (30%) buildings. Cold water (mean 19.3 ± 2.1 °C) is disinfected with sodium hypochlorite, which was detected at points of use (Point I, Point E, and Point T) in a concentration range between 0 and 0.21 mg/L (mean 0.11 ± 0.06 mg/L). In these buildings each flat has an independent boiler for hot water production. Hot water (42.7 ± 12.1 °C) is treated with sodium hypochlorite at concentration between 0 and 0.24 mg/L (mean 0.18 ± 0.09 mg/L) measured at Point A and Point B. Physical-chemical data, measured in hot water samples, showed pH values ranging from 5.7 to 7.3 (mean 6.5 ± 0.9) and conductivity values between 344 and 1089 µS (mean 797 ± 233 µS). Maintenance and cleaning activities of the central water supplies are performed on a half-yearly basis.

On the other hand, all 64 CB have an autoclave system, which distribute municipal water. Only 16 out of 64 (28%) central water supplies are provided with a softener. Sodium hypochlorite is the disinfectant used for cold and hot water treatment and its concentration, detected at points of use, ranged from 0 to 0.1 mg/L (mean 0.04 ± 0.03 mg/L) in cold water (18.6 ± 2.6 °C), and from 0 to 0.15 mg/L (mean 0.07 ± 0.05 mg/L) in hot water (44.2 ± 7.2 °C). All buildings have a thermal central water system with a recirculation device, aimed to energy saving. Physical-chemical data, measured in hot water samples, showed pH values ranging from 5.9 to 7.5 (mean of 6.7 ± 1.1) and conductivity values between 344 and 1081 µS (mean of 799 ± 241 µS). Maintenance and cleaning activities of the cold and hot water systems are performed on a half-yearly basis.

Regarding the TB, all the 35 central water supplies have an autoclave system. Thirty out of 35 (86%) water plumbing distribute municipal water, while five out of 35 (14%) of TB are fed by wells water. No buildings have softeners and an adequate disinfection method. Therefore, all cold water (17.9 ± 2 °C) and hot water (37.4 ± 8.2 °C) samples from TB were not chlorinated. All central water supplies have a boiler device for water storage, heated by solar thermal systems. Physical-chemical data, measured in hot water samples, showed pH values ranging from 5.8 to 7.1 (mean 6.9 ± 1) and conductivity values between 1025 and 1100 µS (mean 1052 ± 115 µS). Maintenance programs results absent in all the TB.

All physical-chemical values regarding the cold and hot water samples, collected at different points of use, are shown in Table 1.

### 3.2. Drinking Water Parameters Results

A variability in microbial growth at 22 °C and 37 °C was observed among the cold-water samples. No significant difference in the total microbial counts at 22 °C and 37 °C was found between samples collected in IB, CB and TB. Overall, the total microbial counts at 22 °C and 37 °C were between 1 and 400 CFU/mL. High bacterial counts (>10^2^ CFU/mL) were detected in 13% (87 out of 660) of the samples, mostly at Point E and Point T.

The total microbial counts at 22 °C and 37 °C detected at the inlet from the aqueduct (Point I) were significantly lower (*p* < 0.0001) than these recorded in samples obtained at the most remote tap from the autoclave (Point T).

In CB and TB, the cold water resulted free from microbiological hazards. Enterococci were not isolated in any sample, but *Serratia liquefaciens* (3 CFU/100 mL) and *Enterobacter cloacae* (4 CFU/100 mL) were isolated at the exit from two different pressure autoclaves.

### 3.3. Legionella spp. Results

*Legionella* spp. was detected in 100 out of 440 (23%) of the hot water samples examined. However, 22 out of 121 (18%) IB, 24 out 64 (38%) CB, and 12 out of 35 (34%) resulted positive to *Legionella*. Overall, 58 out of 220 (26%) examined buildings had at least one sample positive for *Legionella*. A slight increase of colonized buildings was detected in the period between 2014 and 2017 (from 20% to 26%). This may be due to both the increase of the samples number (from 81 in the first year to 220 in all the following three years) and the high percentage of colonized TB, which were analysed only in the period 2016–2017.

In IB *Legionella* positive samples showed counts from 2 × 10^2^ to 4.8 × 10^4^ CFU/L (mean 1 × 10^4^ ± 1.6 × 10^3^ CFU/L). *Legionella pneumophila* sg1, *Legionella pneumophila* sg2–16; and *Legionella* spp. were, respectively, recovered in 12 out of 22 (55%), four out of 22 (18%), and six out of 22 (27%) of the water samples.

**Figure 1:**
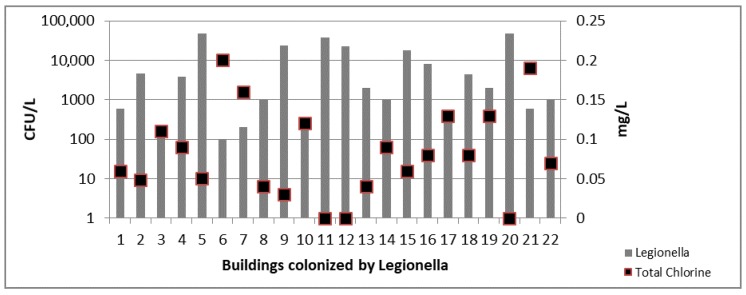
*Legionella* counts and total chlorine concentration detected in 22 colonized IB.

In IB samples statistical results showed moderate correlations between the presence of *Legionella* and the total chlorine concentration decrease (*r* = 0.64; *p* = 0.02) (Figure 1).

In CB *Legionella* positive samples showed counts between 2 × 10^2^ and 3 × 10^4^ CFU/L (mean 5.7 × 10^3^ ± 7.9 × 10^2^ CFU/L). *Legionella pneumophila* sg1, *Legionella pneumophila* sg2–16; and *Legionella* spp. were respectively recovered in nine out of 24 (38%), eight out of 24 (33%), and seven out of 24 (29%) of the water samples.

**Figure 2:**
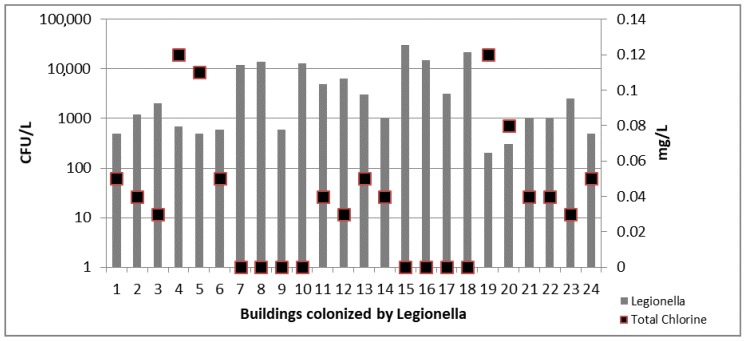
*Legionella* counts and total chlorine concentration detected in 24 colonized CB.

In water samples collected from CB a moderate correlation was detected between the *Legionella* concentration and the reduction of total chlorine values (*r* = 0.67; *p* = 0.022) (Figure 2).

**Figure 3:**
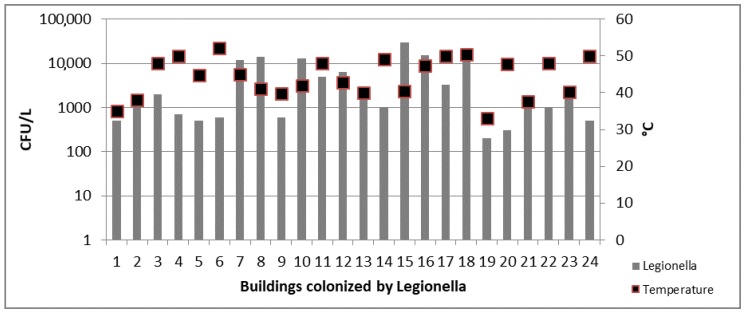
*Legionella* counts and temperature values detected in 24 colonized CB.

Again, in CB samples a weak statistical correlation between the presence of *Legionella* and the increase of the temperature was obtained (*r* = 0.02; *p* = 0.048) (Figure 3).

At last, in TB *Legionella* positive samples showed counts from 3 × 10^2^ to 2.5 × 10^4^ CFU/L (mean 5 × 10^4^ ± 7.1 × 10^2^ CFU/L). *Legionella pneumophila* sg1, *Legionella pneumophila* sg2–16; and *Legionella* spp. were respectively recovered in seven out of 12 (58%), three out of 12 (25%), and two out of 12 (17%) of the water samples.

**Figure 4:**
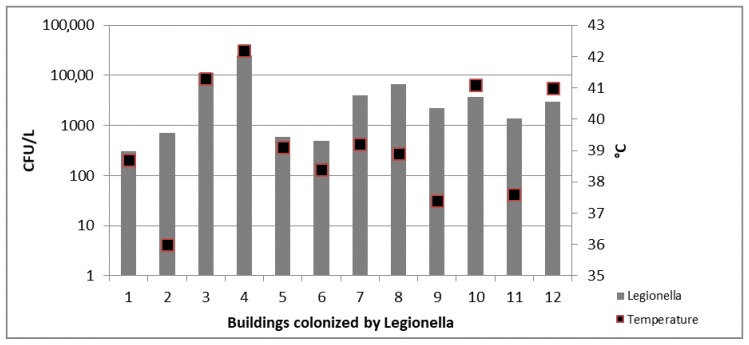
*Legionella* counts and temperature values detected in 12 colonized TB.

In water samples collected from TB a moderate correlation was detected between the *Legionella* concentration and the increase of the temperature (*r* = 0.6; *p* = 0.02) (Figure 4).

## 4. Discussion

Despite water risk control is focused on evaluation of disinfection systems employed to control *Legionella* colonization in hospitals and tourist accommodations water networks, recent global epidemiological data show that almost 80% of the Legionnaire’s disease cases diagnosed in a year are acquired in community [7,8,25]. In Italy, community cases are underestimated for the lack of specific diagnostic tests performed prior to prescribe broad-spectrum antibiotics. In community, the majority of Legionnaire’s disease are considered as atypical pneumonia [20]. This evidence confirms the importance of extending prevention activities and water risk control to residential buildings not associated with a specific outbreak [11].

Home networks contamination represent a real risk for public health, especially for immunocompromised and elderly people. Moreover, European epidemiological data show that at least 50% of *Legionella* infections occurs in people under 65, but a gradual rise of the incidence rates from the age of 40 is documented [8].

Our results, as well as other studies [26,27,28] demonstrate the presence of *Legionella pneumophila* sg1, *Legionella pneumophila* sg2–16, and non-*pneumophila Legionella* species in 26% of the hot water networks analysed, especially in buildings provided with a centralized warm water production system and a solar thermal system for hot water production. In these buildings we detected some moderate correlations between the presence of *Legionella* and the total chlorine concentration decrease and/or the temperature increase. Similar results were obtained in older studies. In particular, from 2004 to 2010 Italian literature data [12,26,27] reveal the presence of *Legionella pneumophila* in small and large residential apartments in Italian cities, with percentages between 22.6% and 34%. As described in our study the highest rate of *Legionella* colonization was detected in hot water networks of CB. In other European countries some slightly lower percentages of flat water networks colonization were obtained (12%) [13,29]. As detected in our study, the European data show some correlations between the *Legionella* growth in pipework and the presence of specific water physical-chemical parameters, as the temperature of 44–48 °C, the absence of chemical disinfection and the pH values around 7.9 [13,29,30].

Another issue evidenced by this study concerns the status of the autoclaves. Statistical data (*p* < 0.0001) show an increase in total microbial counts at 22 °C and 37 °C between the samples collected at the most remote tap from the autoclave compared to the ones directly sampled at the inlet from the aqueduct. Therefore, the higher microbial counts were obtained in water samples collected at distal points of use in apartments. This evidence may be due to sediment formation and debris deposition in the autoclave and in the pipework.

Although our data are similar to those published by other authors [12,13,14,15,16,17] and by our research group [20], this study highlights a *Legionella* related risk in TB, not mentioned in previous studies. This risk is due to the lack of water network maintenance and disinfection. These data provide a more complete evaluation of the *Legionella* hazard in different type of buildings located in the Pisa district.

## 5. Conclusions

In conclusion, our work is the first study evaluating the presence of *Legionella* and the potable water quality in three different types of residential buildings for a 36-month period, proving the need of water system control in residential apartments. The regulations of drinking water quality previously cited in this manuscript extend to building administrators the responsibility of hygienic water control in the building, from the point of delivery by the water supplier up to the points of use. Cold and hot water quality controls must be scheduled using a regular monitoring scheme of water networks to ensure public health safety.

## Figures and Tables

**Table 1:** Mean values of physical-chemical parameters (total chlorine, pH, conductivity, and temperature) detected in hot and cold water sampled at different point of use (Point A, Point B, Point I, Point E, and Point T) of the buildings IB, CB and TB. NA = Not Applied.

Physical-Chemical Parameters	Hot Water Samples		Cold Water Samples		
	Point A	Point B	Point I	Point E	Point T
Building with independent hot water production (IB)					
Total Chlorine (mg/L)	0.21 ± 1.1	0.15 ± 0.07	0.05 ± 0.03	0.17 ± 0.08	0.11 ± 0.05
pH	6.4 ± 0.8	6.6 ± 1.2	NA	NA	NA
Conductivity (µS)	801 ± 253	784 ± 203	NA	NA	NA
Temperature (°C)	48.1 ± 8.6	39.9 ± 15.3	18.7 ± 2.9	19.5 ± 2	19.8 ± 1.4
Building with central hot water production (CB)					
Total Chlorine (mg/L)	0.09 ± 0.07	0.05 ± 0.03	0.03 ± 0.02	0.06 ± 0.03	0.05 ± 0.04
pH	6.6 ± 1	6.7 ± 1.2	NA	NA	NA
Conductivity (µS)	791 ± 235	805 ± 254	NA	NA	NA
Temperature (°C)	48.3 ± 7.9	40.1 ± 7	17.4 ± 2.2	18.9 ± 2.1	16 ± 1.4
Building with solar thermal system for hot water production (TB)					
Total Chlorine (mg/L)	0	0	0	0	0
pH	6.9 ± 1	6.9 ± 1.1	NA	NA	NA
Conductivity (µS)	793 ± 215	799 ± 256	NA	NA	NA
Temperature (°C)	39.5 ± 8.9	33.8 ± 7.9	17.4 ± 2.6	18.9 ± 2.2	17.6 ± 2.9
